# Kinetics of Tyrosinase Inhibitory Activity Using* Vitis vinifera* Leaf Extracts

**DOI:** 10.1155/2017/5232680

**Published:** 2017-06-01

**Authors:** Yung-Sheng Lin, Hui-Ju Chen, Jung-Ping Huang, Pei-Chi Lee, Ching-Ru Tsai, Tzu-Fang Hsu, Wen-Ying Huang

**Affiliations:** ^1^Department of Chemical Engineering, National United University, Miaoli, Taiwan; ^2^Department of Food Science and Biotechnology, National Chung Hsing University, Taichung, Taiwan; ^3^Department of Applied Cosmetology, Master Program of Cosmetic Science, Hung-Kuang University, Taichung, Taiwan

## Abstract

Natural medical plant is considered as a good source of tyrosinase inhibitors. Red vine leaf extract (RVLE) can be applied to a wide variety of medical disciplines, such as treatments for chronic venous insufficiency over many decades. This study investigated the tyrosinase inhibitory activity of RVLE containing gallic acid, chlorogenic acid, epicatechin, rutin, and resveratrol which are effective for skin hyperpigmentation. The five components contents are 1.03, 0.2, 18.55, 6.45, and 0.48 mg/g for gallic acid, chlorogenic acid, epicatechin, rutin, and resveratrol. The kinetic study showed the tyrosinase inhibitory of RVLE via a competitive reaction mechanism. RVLE solution has an IC_50_ (the half inhibitory concentration) value of 3.84 mg/mL for tyrosinase inhibition, that is, an effective tyrosinase inhibitory activity, and can be used as a whitening agent for cosmetic formulations in the future.

## 1. Introduction

A wide variety of skin care products, tagged with effective ingredients for skin hyperpigmentation, become the best-selling products in the cosmetics market across Asia [1]. These products containing extracts of plants, like* Glycyrrhiza uralensis* or* Morus alba*, usually become popular cosmetics. Numerous studies aimed to search for effective materials for skin hyperpigmentation in an effort to reduce dark spots and hyperpigmentation [2–4]. This inhibitory activity of tyrosinase (EC 1.14.18.1) has been extensively studied over the past years [5–7]. Tyrosinase is the rate limiting enzyme of melanogenesis and the main target of antimelanogenesis [8] and catalyzes the oxidation of L-tyrosine to 3,4-dihydroxyphenylalanine (DOPA), which forms dopachrome. These catalyzed reactions result in the formation of melanin, which is responsible for skin pigmentation [9].

Tyrosinase inhibitors can be classified into four types, namely, competitive, uncompetitive, mixed type (competitive/uncompetitive), and noncompetitive inhibitors. Kojic acid shows a typical competitive inhibitory effect of tyrosinase [10].

Studies on tyrosinase inhibitors, such as hydroquinone, azelaic acid, kojic acid, and arbutin, can effectively expand the scope of research on hypermelanosis [11]. However, there exists certain degree of risk when using a great number of well-known tyrosinase inhibitors. For example, dihydroxybenzene may be irritating, mutagenic, and cytotoxic to sensitive skin, while arbutin and kojic acid may result in contact dermatitis and erythema [12]. Therefore, natural medical plants are considered as a good, and alternative, source of tyrosinase inhibitors. Numerous natural tyrosinase inhibitors are identified and reviewed [13, 14].

A red vine leaf is composed of the dried leaves from cultivars of the plant* Vitis vinifera* L. [15]. Red vine leaf extract (RVLE) is herbal medicine involving numerous flavonoids as the major active ingredients thereof [16]. It contains not less than 4% of total polyphenols and 0.2% of anthocyanins [15]. Numerous investigations reported medical applications of RVLE [17], for example, treatments for chronic venous insufficiency over many decades [16, 18]. Besides, it could also improve endurance capacity by facilitating fatty acid utilization in skeletal muscle in mice [19].

Although RVLE has many pharmacological effects, there is no report on the use of RVLE as a tyrosinase inhibitor. Accordingly, this study aims to investigate the kinetics of tyrosinase inhibitory activity using red vine leaf extracts and to develop an alternative natural cosmetic material.

## 2. Materials and Methods

### 2.1. Material

Mushroom tyrosinase, chlorogenic acid, epicatechin, schisandrin, and sodium phosphate monobasic were purchased from Sigma-Aldrich (St. Louis, MO, USA). L-3,4-Dihydroxyphenylalanine (L-dopa) and kojic acid were purchased from Acros (New Jersey, USA). Acetonitrile was purchased from Aencore (Surrey Hills, Australia). Sodium phosphate dibasic anhydrous was purchased from J. T. Baker (Petaling Jaya, Selangor, Malaysia). Methanol was purchased from Merck (Darmstadt, Germany). RVLE (Elastvein®) was purchased from Healthmate (Changhua, Taiwan). Acetic acid was purchased from Panreac (Barcelona, Spain), and gallic acid was purchased from Alfa Aesar (Ward Hill, MA, USA). Rutin was purchased from Extrasynthese (Genay, France). Resveratrol was purchased from TCI (Tokyo, Japan).

### 2.2. Preparation of RVLE Samples

The solvent used for RVLE preparation was deionized water. Two grams of RVLE was sonicated in an ultrasonic bath (Chrom Tech) for 40 min with 98 mL of deionized water. The suspension was centrifuged at 6000 rpm (HERMLE 2206A) for 15 min. The supernatant was collected and run through a 0.45 *μ*m filter. One milligram of schisandrin was dissolved in 10 mL of 70% methanol and then filtered through a 0.45 *μ*m filter. The filtrate was collected as an internal standard solution. Before running the HPLC, 180 *μ*L of the RVLE solution was mixed with 10 *μ*L of the internal standard solution as a sample solution for analysis [20].

### 2.3. Calibration and Validation

Five purchased marker standard solutions, 20 mg of gallic acid, 100 mg of chlorogenic acid, 100 mg of epicatechin, 10 mg of rutin, and 10 mg of resveratrol, were individually dissolved with 10 mL of methanol and stored in a refrigerator for further use. Before adding the internal standard solution, a stock solution was diluted with methanol into a series of standard solutions (gallic acid standard: 60, 30, 15, 7.5, and 5 *μ*g/mL; chlorogenic acid standard: 8.57, 6, 4.29, 3.53, and 3 *μ*g/mL; epicatechin standard: 750, 375, 187.5, 93.75, and 62.5 *μ*g/mL; rutin standard: 100, 50, 33.33, 25, and 20 *μ*g/mL; resveratrol standard: 15, 7.5, 3.75, 1.875, and 1.25 *μ*g/mL). Each solution was analysed twice by HPLC. Peak areas were plotted versus concentrations to establish a calibration curve of each standard. The recovery was determined by comparing the amount of the marker standard added with that of the marker standard found. The detection limit was determined by a signal to noise (S/N) ratio of at least 3 : 1. Relative standard derivation (RSD) for reproducibility derived from the variation of the peak-area ratio or retention time in six replicate samples [21].

### 2.4. HPLC Analysis

An HPLC system (Agilent 1200 Infinity Series, Agilent, USA) is equipped with a quaternary pump, an autosampler, a vacuum degasser, and a diode array detector. A reverse phase column (Cosmosil 5C18-AR II, 5 *μ*m, 25 cm × 4.6 mm ID, Nacalai Tesque, Kyoto, Japan) was used. The mobile phase was a mixture of (A) 0.5% acetic acid and (B) methanol/acetonitrile (2/1, v/v). The mobile phase composition was as follows: 0 min, 90% of (A); 0–10 min, 80% of (A); 10–20 min, 60% of (A); 20–30 min, 40% of (A); and 30–40 min, 0% of (A). The flow rate was 0.8 mL/min, and the wavelength of the detector was set at 280 nm [21].

### 2.5. Analysis of Tyrosinase Inhibitory Activity

Put 40 *μ*L of RVLE solution (3 mg/mL) in a 96-well plate. Then add 40 *μ*L of tyrosinase solutions (0.693 (2.5 U/mL), 1.386 (5 U/mL), 2.772 (10 U/mL), 5.544 (20 U/mL), 6.93 (25 U/mL), and 11.088 (40 U/mL) *μ*g/mL, resp.) in a sodium phosphate buffer at pH 6.8 (PBS) and 120 *μ*L of the 0.625 mM of L-dopa solution (dissolved in PBS). Put another 40 *μ*L of RVLE solution (2, 3, 4, 5, and 6 mg/mL) in a 96-well plate. Then add 40 *μ*L of tyrosinase solution (6.93 *μ*g/mL) and 120 *μ*L of 0.625 mM of L-dopa solution. These mixed solutions were kept at 37°C for 30 min to find the suitable concentrations of tyrosinase used for the tyrosinase inhibitory activity of RVLE. The absorbance was measured at 475 nm [22] using a Microplate-Reader (Sunrise Basic, Grödig, Austria). Kojic acid (0.01, 0.02, 0.04, 0.06, and 0.08 mg/mL) was used as a positive control, and the solvent control used as a blank was deionized water. The tyrosine inhibition (%) was calculated by

(1) $$\begin{array}{c} The\;\;inhibition\;\;rate\left(\;\%\;\right)=\left(\;1-\frac{\Delta OD_{sample}}{\Delta OD_{control}}\;\right)\times 100\%, \end{array}$$

where ΔOD_sample_ and ΔOD_control_ represent the absorbance of the sample and the control measured at 475 nm, respectively. An IC_50_ (the half inhibitory concentration) value was determined by regression of a dose-response curve at which 50% target activity was lost.

### 2.6. Kinetic Properties of RVLE

A Lineweaver-Burk plot was made by plotting the inverse numbers of the reaction rate *V* and the concentration of substrate [S]:

(2) $$\begin{array}{c} \frac{1}{V}=\frac{K_{m}}{V_{max}}\times \frac{1}{\left[S\right]}+\frac{1}{V_{max}}. \end{array}$$

A linear regression model created in a double reciprocal plot can be used to determine the Michaelis constant *K*_*m*_ and the maximum velocity *V*_max_, for the reason that the curves therein have the *x*-intercept 1/*K*_*m*_, the *y*-intercept 1/*V*_max_, and the slope of *K*_*m*_/*V*_max_. In addition, a Lineweaver-Burk plot can be used to rate the performance of an inhibitor as competitive, noncompetitive, or even uncompetitive [23].

Before the kinetic studies, the dose-dependent relationship between RVLE and tyrosinase was checked first. Put 40 *μ*L of RVLE solution (0.1875, 0.375, 0.75, 1.5, and 3 mg/mL) in a 96-well plate. Then add 40 *μ*L of tyrosinase solutions (0.693, 1.386, 2.772, 5.544, and 11.088 *μ*g/mL). The substrate was L-dopa by 120 *μ*L 0.1 mM in a sodium phosphate buffer at pH 6.8.

For kinetic studies, another 40 *μ*L of RVLE solution (0, 0.375, 0.75, 1.5, and 3 mg/mL) was put in a 96-well plate, and then 40 *μ*L of tyrosinase solutions (2.772 *μ*g/mL) was added. The substrate was L-dopa solution which was made by 120 *μ*L of dissolving L-dopa (0.078, 0.1, 0.156, 0.3125, and 0.625 mM, resp.) in a sodium phosphate buffer at pH 6.8. A Lineweaver-Burk plot was made by plotting the inverse numbers of the reaction rate (*V*) and the concentration of L-dopa (2) [23].

### 2.7. Statistical Analysis

Statistical evaluation was performed by running one-way analysis of variance (ANOVA) with SASR software (version 6.08, SAS Institute Inc., Cary, NC, USA). All data were presented as means and standard deviations (mean (SD)). Differences were considered statistically significant in case of a *p* value less than 0.05.

## 3. Results and Discussion

### 3.1. HPLC Analysis

**Figure 1:**
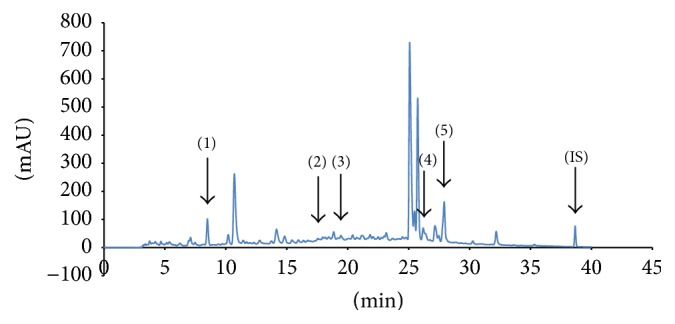
HPLC chromatograms of RVLE solution: (1) gallic acid, (2) chlorogenic acid, (3) epicatechin, (4) rutin, and (5) resveratrol and (IS) schisandrin.

Figure 1 shows the representative HPLC chromatograms of RVLE solution. The bioactive components of RVLE solution are gallic acid, chlorogenic acid, epicatechin, rutin, and resveratrol, which were effective compounds for skin hyperpigmentation confirmed by previous studies [24–27]. These components were identified by a comparison of HPLC chromatograms with standards. Based on the chromatographic analysis results, the five components contents are 1.03, 0.2, 18.55, 6.45, and 0.48 mg/g for gallic acid, chlorogenic acid, epicatechin, rutin, and resveratrol, respectively.

### 3.2. Inhibitory Ability of RVLE Solution

**Figure 2:**
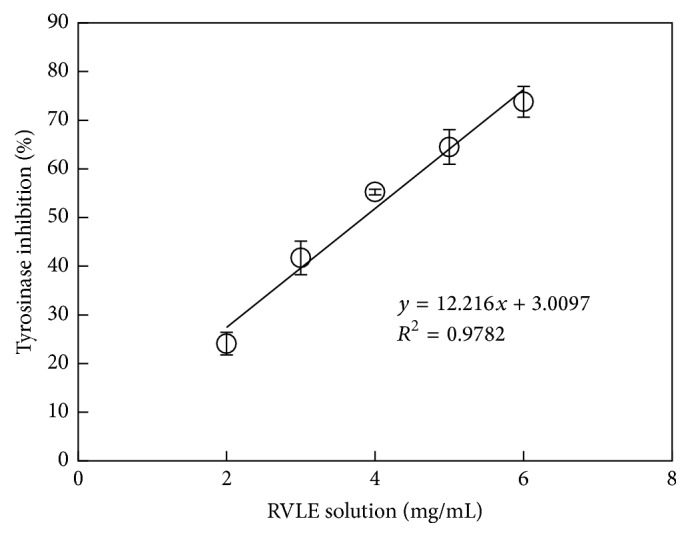
Inhibition of the tyrosinase activity using RVLE solution as an inhibitor (40 *μ*L of tyrosinase solution (6.93 *μ*g/mL) and 120 *μ*L of 0.625 mM of L-dopa solution were added for each measurement).

**Figure 3:**
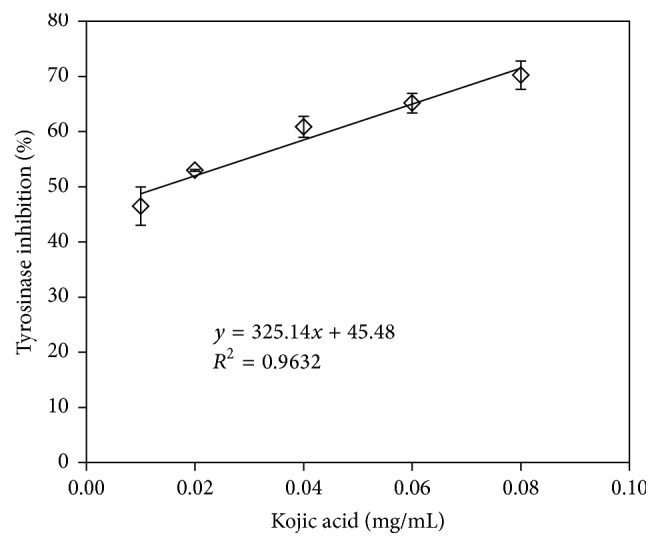
Inhibition of the tyrosinase activity using kojic acid as an inhibitor.

Figure 2 shows the inhibition of tyrosinase activity using RVLE solution as an inhibitor. RVLE solution reduced the tyrosinase activity in a dose-dependent manner. The linear regression line has a slope of 12.216 and *y*-intercept of 3.0097. The IC_50_ value of RVLE solution was evaluated as 3.84 mg/mL. For comparison, kojic acid was used as a positive control in this study. As in Figure 3, the inhibition of tyrosinase activity increased with the added amount of kojic acid, and its IC_50_ was 0.014 mg/mL. The tyrosinase inhibitory activity of* Vitis vinifera* L. was reported in the previous study using the extract of dried stems of the grape tree [28]. Although RVLE solution does not outperform kojic acid as an inhibitor, it was still generally recognized as a safe natural ingredient and could be safely used in cosmetic products [29].

### 3.3. Kinetic Study of Tyrosinase Inhibitory Activity

**Figure 4:**
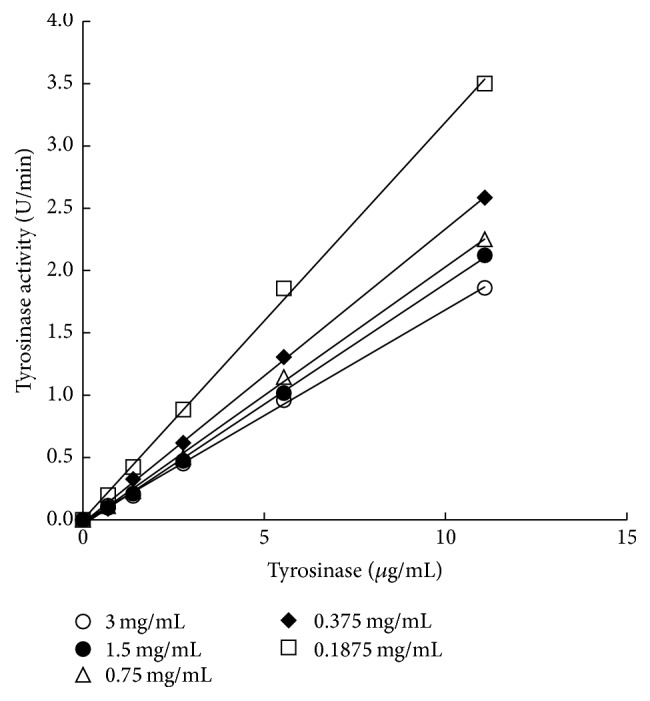
Influence of the RVLE concentration on the tyrosinase activity with 0.1 mM of L-dopa as a substrate.

RVLE solution showed the ability to inhibit the formation of dopachrome, which can be detected using a spectrophotometer at a wavelength of 475 nm. With 0.1 mM of L-dopa as a substrate, tyrosinase activity increased with the added amount of tyrosinase. As illustrated in Figure 4, there exists a linear relationship between the tyrosinase activity and the tyrosinase concentration, while the tyrosinase activity decreases with the added amount of RVLE solution. The RVLE solution is hence validated to inhibit tyrosinase activity successfully. In addition, low slope curves were seen as the amount of RVLE solution increased.

**Figure 5:**
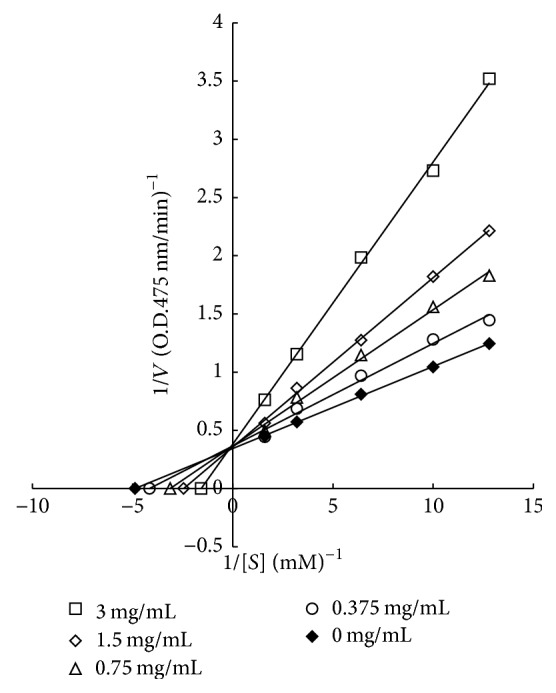
Lineweaver-Burk double reciprocal plot of RVLE solution with the concentration as a parameter (*V*: absorbance change rate, ΔOD_475 nm_/min; [*S*]: concentration of L-dopa).

The inhibitory mechanism of RVLE could be further investigated by means of this kinetic study.  Figure 5 shows a Lineweaver-Burk double reciprocal plot with the concentration of RVLE solution as a parameter. With L-dopa as a substrate, the curves share the same *y*-intercept 1/*V*_max_, while the *x*-intercept −1/*K*_*m*_ increased with the concentration of RVLE solution, as illustrated in Figure 5. In other words, *V*_max_ = 0.36 mM for all cases, but *K*_*m*_ increased due to the introduction of the inhibitor. In this context, the RVLE binding by tyrosinase had an effect on the L-dopa binding. Therefore, L-dopa and RVLE bound at the same sites on the tyrosinase. According to Figure 5, the inhibitory activity was rated as competitive.

## 4. Conclusions

In this study, it was concluded that a red vine leaf extract (RVLE) solution successfully reduced the tyrosinase activity. It provided an IC_50_ value of 3.84 mg/mL for tyrosinase inhibition, and the tyrosinase inhibitory activity was rated as competitive. The bioactive components of RVLE solution contained gallic acid, chlorogenic acid, epicatechin, rutin, and resveratrol. Therefore, RVLE solution could be used in cosmetic formulations as a natural whitening agent.
